# Investigation of Biofilm Formation and Antimicrobial Resistance in Bacteria Isolated from Hospital Medical Devices

**DOI:** 10.3390/microorganisms14071429

**Published:** 2026-06-30

**Authors:** Ilaria Cosimato, Giuseppe Di Siervi, Mariagrazia De Prisco, Federica Dell’Annunziata, Nicoletta Capuano, Noemi Cafà, Anna Barbato, Josè Camilla Sammartino, Flora Salzano, Pasquale Pagliano, Giovanni Boccia, Francesco De Caro, Giuseppe Rescigno, Gianluigi Franci

**Affiliations:** 1 Department of Medicine, Surgery and Dentistry “Scuola Medica Salernitana”, University of Salerno, 84081 Baronissi, Italy; ilaricos96@gmail.com (I.C.); giuseppedisiervi200@gmail.com (G.D.S.); depriscomariagrazia22@gmail.com (M.D.P.); niccapuano@unisa.it (N.C.); cafanoemi@gmail.com (N.C.); flsalzano@unisa.it (F.S.); ppagliano@unisa.it (P.P.); gboccia@unisa.it (G.B.); fdecaro@unisa.it (F.D.C.); 2 U.O.S. Microbiology and Virology, AOU San Giovanni di Dio e Ruggi d’Aragona, Via San Leonardo, 84131 Salerno, Italy; f.dellannunziata@unilink.it (F.D.); anna.barbato@sangiovannieruggi.it (A.B.); 3 Department of Life Science, Health, and Health Professions, Link Campus University, 00165 Rome, Italy; 4 Department of Clinical-Surgical, Diagnostic and Pediatric Sciences, Università degli Studi di Pavia, 27100 Pavia, Italy; josecamilla.sammartino@unipv.it; 5 U.O.C. Infectious Disease, AOU San Giovanni di Dio e Ruggi d’Aragona, Via San Leonardo, 84131 Salerno, Italy; 6 U.O.C. Hospital and Epidemiological Hygiene, San Giovanni di Dio e Ruggi D’Aragona, Via San Leonardo, 84131 Salerno, Italy; 7 U.O.C. Risk Assessment Management and Reporting, San Giovanni di Dio e Ruggi d’Aragona, 84131 Salerno, Italy; 8 U.O.C. Clinical Pathology, San Giovanni di Dio e Ruggi d’Aragona, 84131 Salerno, Italy; g.rescigno@sangiovannieruggi.it

**Keywords:** medical device, biofilm, device, MDR biofilm, catheter and stent

## Abstract

**Background:** Medical device-associated infections represent a major component of healthcare-associated infections. Biofilm formation promotes microbial persistence on device surfaces, reduces antimicrobial susceptibility, and contributes to multidrug resistance (MDR), complicating diagnosis and treatment. **Materials and Method:** This study investigated biofilm production and antimicrobial resistance in microorganisms recovered from 100 indwelling and implantable medical devices, including urinary and venous catheters, urethral stents, catheter tips, and orthopedic or prosthetic materials, collected at a tertiary-care hospital (AOU “San Giovanni di Dio e Ruggi d’Aragona”, Salerno, Italy). Microbiological cultures were performed using direct and enrichment methods. Microbial identification was carried out by MALDI-TOF MS, antimicrobial susceptibility testing by VITEK^®^ (bioMérieux, Marcy-l'Étoile, France) 2 according to EUCAST criteria, and biofilm production was assessed using the crystal violet tissue culture plate assay. MDR status was defined according to international guidelines. **Results:** Microbial growth was detected in the majority of analized devices, frequently with polymicrobial contamination. Within the study cohort, coagulase-negative staphylococci (CoNS) were the most frequently recovered microorganisms (20%), followed by *Klebsiella pneumoniae* (10%), *Candida albicans* (9%), *Staphylococcus aureus* (9%), *Enterococcus faecalis* (8%), and *Escherichia coli* (8%). A significant association was observed between multidrug resistance and biofilm production, with MDR isolates showing a markedly higher likelihood of being biofilm producers compared with non-MDR isolates (OR 9.50; 95% CI 2.72–42.96; *p* < 0.005). Biofilm formation also differed significantly among device types (*p* = 0.028). **Conclusions:** These findings indicate a high prevalence of biofilm-producing MDR microorganisms among isolated recovered from medical devices in our cohort and highlight a significant association between MDR phenotype and biofilm production. These results provide a microbiological characterization of device-associated isolates that may support future studies on infection dynamics and control strategies.

## 1. Introduction

Biofilms are structured communities of microorganisms embedded in a self-produced extracellular polymeric matrix. Following initial attachment, bacteria progressively establish stable adhesion to a surface, which may consist of biological tissue or an inert abiotic material. Biofilm formation is tightly regulated by quorum sensing, a cell–cell communication process that enables bacteria to synchronously respond to external and internal signals by modulating gene expression as a function of cell density [1].

Infections associated with biofilm formation represent a major complication in hospitalized patients, particularly in those carrying indwelling medical devices such as central venous catheters, urinary catheters, and other implanted materials [2]. Biofilm development on device surfaces can lead to persistent and difficult-to-treat infections, increasing the risk of long-term complications and prolonged hospitalization time. In addition, biofilms play a pivotal role in antimicrobial tolerance and resistance by reducing cell permeability, enhancing efflux pump activity, and promoting the production of antibiotic-modifying enzymes [3]. These mechanisms make bacterial eradication particularly challenging and complicate therapeutic management [4].

It has been estimated that approximately 50–70% of healthcare-associated infections are associated with indwelling medical devices, representing a major burden for modern healthcare systems [5,6]. Mortality associated with these infections largely depends on the type of implanted device [7]. Among device-associated infections, central line-associated bloodstream infections (CLABSIs) and catheter-related bloodstream infections (CRBSIs) are associated with particularly high morbidity, mortality, and healthcare costs. These infections are most commonly caused by biofilm-forming microorganisms, including coagulase-negative staphylococci (CoNS), *Staphylococcus aureus*, *Enterococcus* spp., *Klebsiella pneumoniae*, *Escherichia coli*, *Pseudomonas aeruginosa*, *Acinetobacter baumannii*, and *Candida* spp. [8] Gram-positive bacteria, particularly *Staphylococcus aureus* and coagulase-negative staphylococci (CoNS), are among the main pathogens responsible for these infections. Other less common, yet clinically relevant, pathogens include *E. coli*, *Enterococcus* spp., and *Candida* spp. [9]. Although *E. coli* is commonly associated with catheter-associated urinary tract infections, it accounted for only a minority of the isolates recovered from urinary devices in the present study. This discrepancy likely reflects the specific composition of the analyzed medical device cohort and highlights the variability of microbial distribution across different device types. Overall, these microorganisms readily colonize both abiotic and biotic surfaces, forming biofilms that are highly recalcitrant to antimicrobial therapy. Among them, *P. aeruginosa* is particularly relevant, as it is often linked to severe complications such as sepsis and increased mortality in hospitalized patients.

Emerging evidence suggests a close relationship between biofilm formation and antimicrobial resistance, as both mechanisms contribute to microbial persistence on device surfaces and complicate eradication [3]. However, although an association between antimicrobial resistance and biofilm formation has been increasingly reported, the extent to which multidrug resistance (MDR) independently contributes to enhanced biofilm production remains to be fully elucidated, particularly in heterogeneous collections of clinical isolates recovered from different medical devices.

Based on this evidence, we hypothesized that microorganisms exhibiting an MDR phenotype would display an enhanced biofilm-forming capacity compared with non-MDR isolates. Therefore, the aim of this study was to characterize the distribution of microorganisms colonizing a broad panel of indwelling and implantable medical devices (including urinary and venous catheters, urethral stents, catheter tips, and prosthetic or fixation materials) explanted from patients in a single tertiary-care hospital and to investigate the association between MDR phenotype and biofilm production in these device-associated clinical isolates, in a setting that includes a broader variety of devices and microbial species than most previous reports.

## 2. Materials and Methods

### 2.1. Specimen Collection

From January 2025 to June 2025, a total of 100 explanted medical devices were collected at the San Giovanni di Dio e Ruggi D’Aragona Hospital (Salerno, Italy). The devices comprised 45 bladder catheters, 16 venous catheters, 7 urethral stents, 5 atrial catheters, 5 bladder catheter tips, 4 femoral catheters, 4 screws, 2 catheter tips, 2 ventricular catheters, 2 central venous catheters, 2 central venous catheter tips, and one device each of the following: articular catheter, umbilical catheter, clamp, aortic valve prosthesis, cephalic screw, and distal screw.

All specimens were collected under sterile conditions, transported to the bacteriology laboratory, and processed for microbiological analysis immediately upon arrival.

### 2.2. Medical Device Culture and Isolates Identification

Different microbiological protocols were applied according to device type. Catheter-associated devices, including urinary and venous catheters, were aseptically sectioned into two fragments using sterile forceps. One fragment was directly rolled onto Columbia agar supplemented with 5% sheep blood (bioMérieux, Marcy l’Étoile, France) and incubated in a CO_2_-enriched atmosphere. The second fragment was inoculated into 10 mL of Brain Heart Infusion (BHI, bioMérieux, Marcy l’Étoile, France) broth and incubated for 24 h at 37 °C. Subsequently, broth cultures were subcultured onto Columbia Colistin-Nalidixic Acid (CNA, bioMérieux, Marcy l’Étoile, France) agar, MacConkey agar (bioMérieux, Marcy l’Étoile, France), Sabouraud glucose agar (bioMérieux, Marcy l’Étoile, France), and chocolate agar (bioMérieux, Marcy l’Étoile, France). Plates were incubated at 37 °C and examined after 24 and 48 h.

Prosthetic materials and fixation devices (e.g., prostheses and screws) were processed using a standardized sonication protocol to improve microbial recovery. Briefly, each specimen was placed in a sterile container filled with saline solution covering at least 90% of its volume. Samples were vortexed for 30 s, sonicated in a pre-degassed water bath (30–40 kHz; 0.22 ± 0.04 W/cm^2^) for 5 min, and vortexed again for 30 s.

After sonication, 0.5 mL of sonicate was plated onto blood agar and chocolate agar and incubated at 36 ± 1 °C in 5% CO_2_ for up to 5 days. In parallel, 10 mL of sonicate was inoculated into aerobic and anaerobic blood culture bottles and incubated in an automated system for up to 7 and 14 days (aerobic) and 14 days (anaerobic). In case of culture positivity, 10 µL aliquots from each bottle were subcultured onto the same selective and differential media described above for catheter-derived samples.

Microbial identification was performed using matrix-assisted laser desorption/ionization time-of-flight mass spectrometry (MALDI-TOF MS; bioMérieux, Marcy l’Étoile, France), according to the manufacturer’s instructions.

### 2.3. Antimicrobial Susceptibility Testing

Antimicrobial susceptibility testing (AST) was carried out using the VITEK^®^ 2 automated system (bioMérieux, Marcy l’Étoile, France). Bacterial suspensions were prepared in saline solution and adjusted to 0.5 McFarland using a densitometer. Appropriate AST cards were selected based on the organism type (Gram-positive, Gram-negative, *Enterococcus* spp., and yeasts) and processed using the automated system.

The system provided minimum inhibitory concentration (MIC) values and categorical interpretations (susceptible, intermediate, resistant) according to EUCAST breakpoints.

The antimicrobial panel included representatives of the main pharmacological classes: β-lactams (e.g., ampicillin, piperacillin/tazobactam, cephalosporins, carbapenems), aminoglycosides, fluoroquinolones, tetracyclines, glycopeptides, lipopeptides, oxazolidinones, macrolides, and other agents such as fosfomycin, rifampicin, and colistin. Antifungal agents included azoles, echinocandins, amphotericin B, and flucytosine. MDR was defined as non-susceptibility to at least one agent in three or more antimicrobial classes. Based on this definition, isolates were classified as MDR (*n* = 59) or non-MDR (*n* = 41) prior to statistical analysis. Comparative analyses were performed to evaluate the association between MDR status and biofilm-forming capacity.

#### Biofilm Formation Assays

Biofilm formation was assessed using the tissue culture plate (TCP) assay. Briefly, bacterial cells were grown in Luria–Bertani (LB; Sigma-Aldrich, Oxoid, Basingstoke, NH, USA) broth supplemented with 1% glucose and incubated at 37 °C for 24 h. Following incubation, cultures were adjusted to an optical density at 600 nm of 0.2 (approximately 10^8^ CFU/mL) using fresh glucose-supplemented LB medium.

A volume of 100 μL of each standardized suspension was dispensed into 96-well flat-bottom microtiter plates (Thermo Fisher Scientific, Burlington MA, USA) and incubated statically at 37 °C for 24 h. Wells containing sterile medium without bacterial cells were included as negative controls. *S. aureus* ATCC 6538 and *S. aureus* ATCC 25923 were used as negative and positive controls for biofilm production, respectively.

After incubation, wells were gently washed twice with phosphate-buffered saline (PBS) to remove non-adherent planktonic cells, air-dried and stained with 0.1% crystal violet (CV) (Sigma-Aldrich, St. Louis, MO, USA) for 40 min at RT. Excess stain was removed by washing three times with PBS, and the bound dye was solubilized with 95% ethanol for 20 min under orbital shaking at RT. Biofilm biomass was quantified by measuring absorbance at 570 nm using a microplate reader (Tecan Infinite M200 Pro, Tecan Group Ltd., Männedorf, Switzerland). The cut-off value (ODcutoff) was calculated as the mean OD of the negative control plus three standard deviations. Biofilm production was classified as follows:

ODcutoff = ODavg of negative control + (3 × standard deviation of ODs of negative control).

The ODcutoff criterion and the corresponding classification of isolates as non-, weak, moderate-, or strong biofilm producers were adopted according to the methodology originally described by Stepanović et al. and subsequently validated and applied in numerous studies using crystal violet-based microtiter plate assays. This approach provides a statistically defined threshold above background staining, allowing discrimination between non-adherent and biofilm-forming isolates [10,11,12].

Biofilm-forming capacity was categorized according to the following criteria

(i) OD ≤ ODcutoff: non-biofilm producer
(ii) ODcutoff < OD ≤ 2 × ODcutoff: weak biofilm producer
(iii) 2 × ODcutoff < OD ≤ 4 × ODcutoff: moderate biofilm producer
(iv) OD > 4 × ODcutoff: strong biofilm producer

All experiments were performed in triplicate.

### 2.4. Statistical Analysis

Statistical analysis was conducted using R software (version 4.3.3; R Foundation for Statistical Computing, Vienna, Austria) [13]. Descriptive statistics were used to summarize the distribution of medical devices and the corresponding microorganisms isolated. Prior to statistical analyses, data were assessed for compliance with the assumptions required for parametric testing. Normality was evaluated using the Shapiro–Wilk test, whereas homogeneity of variances was assessed before applying parametric tests. To assess whether a statistically significant association existed between being MDR status and biofilm forming ability, Fisher’s exact test was applied. A t-test was used to compare mean biofilm production between MDR and non-MDR groups, whereas one-way ANOVA was employed to compare mean OD values across device type. A *p*-value < 0.05 was considered statistically significant.

## 3. Results

### 3.1. Microorganism Distribution in Different Medical Devices

**Figure 1:**
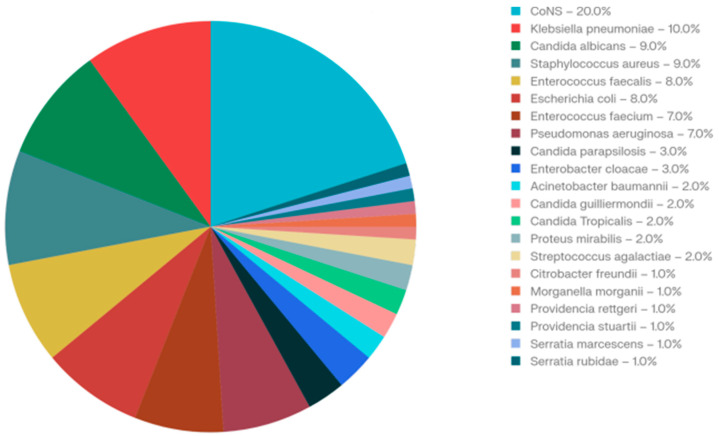
Distribution of microorganisms isolated from colonized medical devices. Each slice represents the relative abundance, expressed as percentage, of each species isolated across all medical devices analyzed. All bacterial species identified are reported color-coded and with their relative percentage in the legend.

Within the collection of medical devices analyzed in the present study, microbial colonization was predominantly sustained by coagulase-negative staphylococci (CoNS, 20%), followed by *K. pneumoniae* (10%), *C. albicans* (9%), and *S. aureus* (9%). These species displayed the highest relative frequencies across catheters, stents, and catheter tips, indicating that they constitute the main contributors to device-associated contamination in the cohort studied (Figure 1).

### 3.2. Mono-, Bi-, and Trimicrobial Contamination

**Figure 2:**
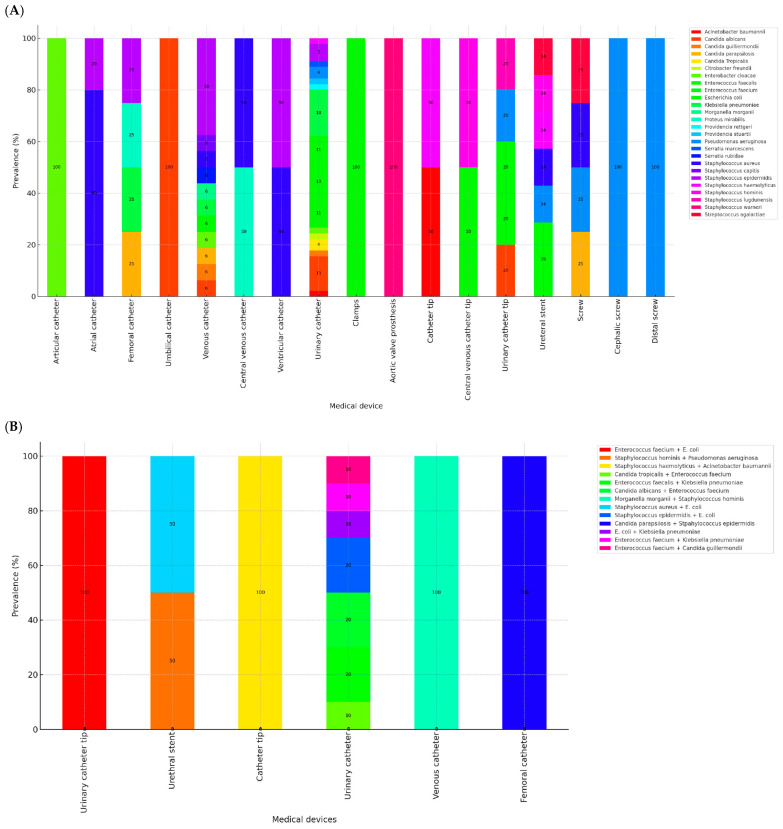

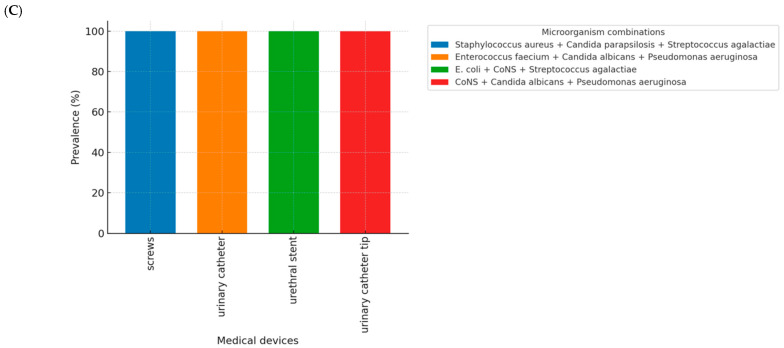
Bacterial and fungal contamination across medical devices. Monomicrobial (**A**) contamination is the most represented across all medical devices, the number of analyzed devices for each category was as follows: 45 bladder catheters, 16 venous catheters, 7 urethral stents, 5 atrial catheters, 5 bladder catheter tips, 4 femoral catheters, 4 screws, 2 catheter tips, 2 ventricular catheters, 2 central venous catheters, 2 central venous catheter tips, 1 articular catheter, 1 umbilical catheter, 1 clamp, 1 aortic valve prosthesis, 1 cephalic screw, and 1 distal screw, followed by bimicrobial (**B**) contamination that occurred in 6 types of medical devices and trimicrobial (**C**) contamination that was the rarest, occurring only in screws and urinary tract related medical devices. The bacterial and fungal species identified are reported in the legends (color-coded) and in relative percentage of isolates identified per medical device analyzed.

The distribution of contamination levels across medical devices, stratified as mono-, bi-, or trimicrobial, is reported in Figure 2. For each device type, microbial distribution was expressed as the percentage of isolates represented by each species relative to the total number of microorganisms recovered from that specific category. All medical device types showed at least monomicrobial contamination. Bimicrobial contamination was observed in a limited number of devices (n = 6) and showed heterogeneous species combinations. For example, the urinary catheter tip yielded a single combination (*E. faecium* + *E. coli*), whereas urinary catheters and urethral stents showed a greater diversity of combinations (Figure 2B). These findings should be interpreted in the context of the limited number of observations for some device categories.

Trimicrobial contamination was less frequent and observed only in selected devices, including screws and urinary tract-related devices (Figure 2C).

### 3.3. Resistance Profiles of Microorganisms

#### 3.3.1. Gram-Positive Bacterial Resistance

Among Gram-positive isolates, the highest resistance rates to β-lactams were observed for benzylpenicillin and oxacillin, particularly in *S. aureus*, and in five species of *S. aureus* and several CoNS species (*S. capitis*, *S. epidermidis*, *S. haemolyticus*, *S. hominis*, *and S. lugdunensis*), with values reaching up to 100% in some cases. However, these estimates should be interpreted with caution, as they are based on a limited number of isolates.

A substantial proportion of staphylococcal strains also showed resistance to clindamycin and erythromycin, whereas linezolid and vancomycin retained low resistance rates across most staphylococcal species.

Enterococcal isolates displayed a distinct resistance profile: *Enterococcus faecium* showed high resistance percentages to ampicillin and amoxicillin/clavulanic acid (up to 100%), as well as elevated resistance to imipenem, kanamycin, and streptomycin, whereas *E. faecalis* showed comparatively lower resistance rates. Resistance to linezolid and vancomycin remained limited (Table 1). Overall, *E. faecium* exhibited resistance to a broader range of antimicrobial classes compared with *E. faecalis*.

For *Streptococcus agalactiae*, high resistance rates were observed for erythromycin and clindamycin, whereas resistance to other antibiotics remained comparatively low.

#### 3.3.2. Gram-Negative Bacterial Resistance

Among Gram-negative isolates, non-fermenting bacilli and selected Enterobacterales displayed the most concerning resistance profiles.

*A. baumannii* exhibited resistance rates of 100% to multiple antibiotics, including amoxicillin/clavulanic acid, ciprofloxacin, gentamicin, levofloxacin, meropenem, and trimethoprim/sulfamethoxazole, with lower resistance observed for colistin and piperacillin/tazobactam.

*P. aeruginosa* displayed high resistance (83.3%) to cefepime, ceftazidime, and ciprofloxacin, together with relevant resistance to fosfomycin, gentamicin and piperacillin/tazobactam.

Among Enterobacterales, *Citrobacter freundii* and *Providencia stuartii* showed high resistance percentages to the reported β-lactams, including third- and fourth-generation cephalosporins and the newer combinations ceftazidime/avibactam and ceftolozane/tazobactam. *K. pneumoniae* exhibited resistance rates of around 80% to amoxicillin/clavulanic acid, cefepime, and ceftazidime, with variable but consistently elevated resistance to ceftazidime/avibactam, fosfomycin, meropenem, and piperacillin/tazobactam. *E. coli* exhibited generally lower resistance rates (around 50% for selected antibiotics), with reduced resistance to carbapenems and colistin.

Less frequent species (e.g., *Morganella morganii*, *Proteus mirabilis*, *Providencia rettgeri*, and *Serratia* spp.) also contributed to the resistance landscape. Although resistance rates of up to 100% were observed for selected antibiotics, these estimates were derived from a small number of isolates and should therefore be interpreted cautiously.

#### 3.3.3. Antifungal Resistance Profiles of Yeast Isolates

Regarding yeasts, *C. albicans* showed non-negligible resistance rates to fluconazole (22.2%), micafungin (11.1%), and voriconazole (22.2%). *C. parapsilosis* exhibited higher resistance to fluconazole and voriconazole (33.3% each), suggesting reduced susceptibility to azoles in the analyzed sample.

### 3.4. Prevalence of Biofilm-Producing Strains

**Figure 3:**
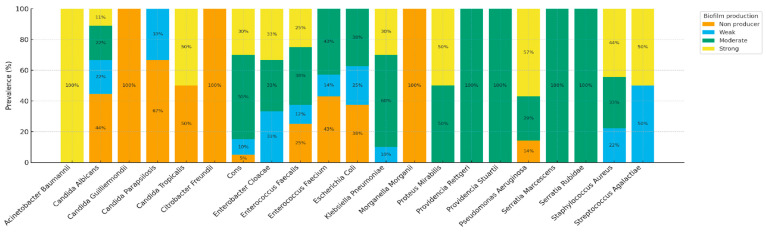
Bacterial prevalence based on different biofilm production.

Microbial isolated exhibited heterogeneous biofilm-forming capacity. Based on quantitative assessment, isolates were classified as weak, moderate, or strong producers, allowing evaluation of species-specific biofilm behavior (Figure 3).

### 3.5. Resistance Profile of Gram-Positive MDR Biofilm-Producing Versus Non-MRD Biofilm Non-Producing Bacteria

**Figure 4:**
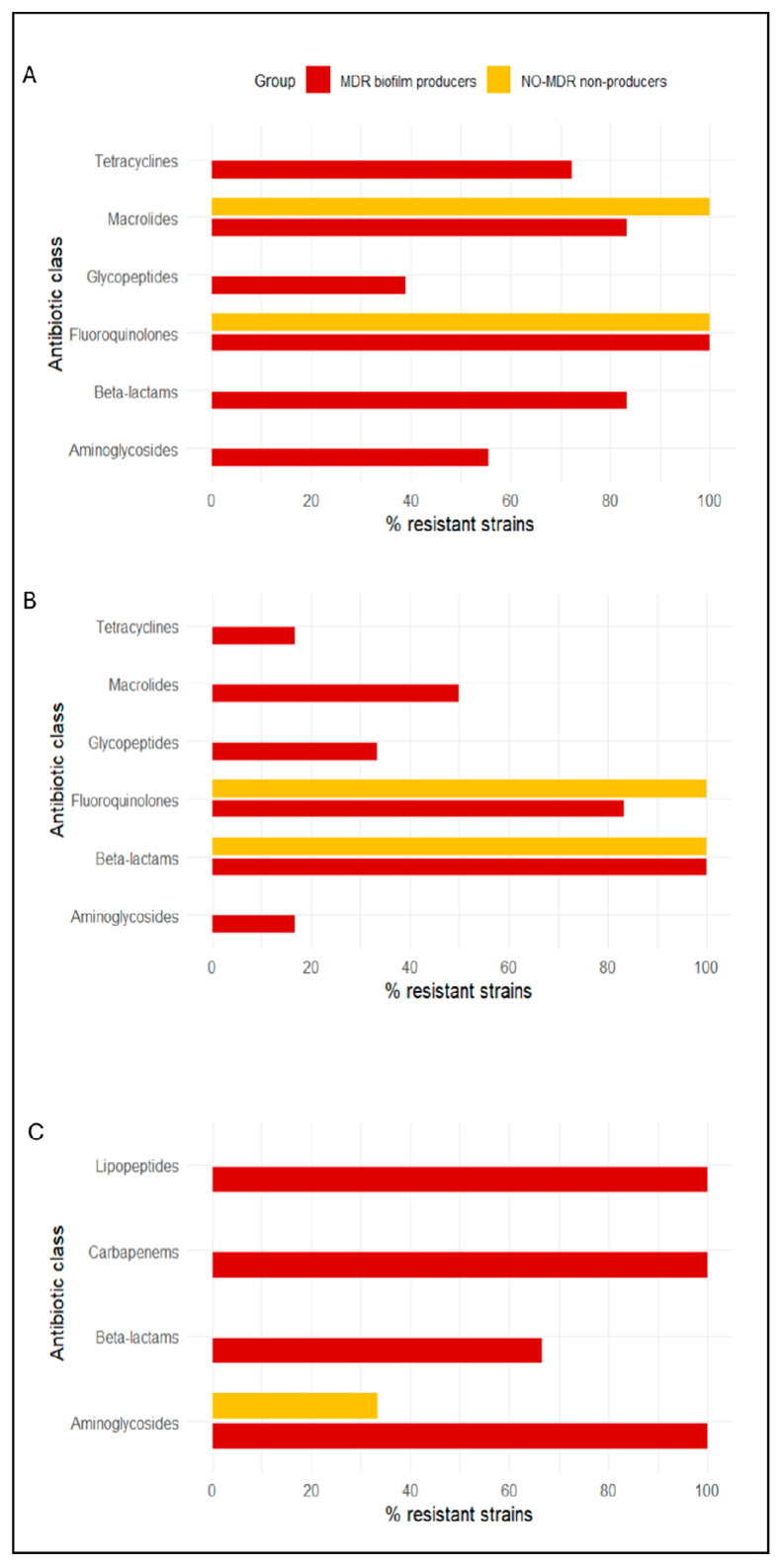
Antibiotic class resistance profile for the main Gram-positive bacteria stratified by MDR status and biofilm production. (**A**) Coagulase-negative staphylococci (CoNS), (**B**) *Staphylococcus aureus*, (**C**) *Enterococcus faecalis*.

Isolates were stratified into two extreme phenotypic groups in order to assess the potential clinical impact associated with the coexistence of multidrug resistance and biofilm-forming capacity: MDR biofilm-producing isolates and non-MDR non-biofilm-producing isolates. The purpose of this comparison was not to establish a complete phenotypic classification of all isolates, but to highlight the most clinically relevant and the least clinically concerning profiles. The analyses showed that MDR biofilm-producing CoNS exhibit broad resistance across multiple antibiotic classes compared with non-MDR non-biofilm-producing isolates. Aminoglycosides also display intermediate-to-high resistance levels, whereas glycopeptides, although still partially effective, show a non-negligible degree of resistance. In the latter group, resistance was mainly confined to macrolides and fluoroquinolones, in some cases with higher percentages for these classes compared with MDR biofilm-producing isolates (Figure 4A).

A similar pattern was observed for *S. aureus* (Figure 4B). MDR biofilm-producing strains displayed very high resistance rates to β-lactams and fluoroquinolones, intermediate levels to macrolides and glycopeptides, and lower rates to aminoglycosides and tetracyclines. In non-MDR non-biofilm-producing strains, resistance was mostly concentrated in β-lactams and fluoroquinolones, with percentages that in some cases exceed those observed in MDR biofilm-producing isolates (Figure 4B). Regarding *E. faecalis*, MDR biofilm-producing strains exhibited high resistance across several classes, with some values approaching 100%, whereas non-MDR non-biofilm-producing isolates exhibited resistance primarily to aminoglycosides (Figure 4C).

### 3.6. Resistance Profile of Gram-Negative MDR Biofilm-Producing Versus Non-MRD Non-Producing Bacteria

**Figure 5:**
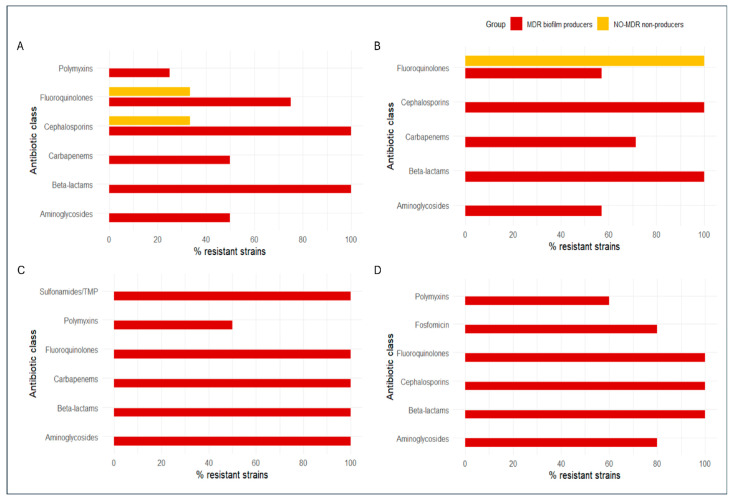
Antibiotic class resistance profile for the main Gram-negative bacteria stratified by MRD status and biofilm production. (**A**) *Escherichia coli*, (**B**) *Klebsiella pneumoniae*, (**C**) *Acinetobacter baumannii*, (**D**) *Pseudomonas aeruginosa*.

In MDR biofilm-producing *E. coli*, resistance reached high levels for β-lactams and cephalosporins, in some cases up to 100%, with only slightly lower rates for fluoroquinolones, carbapenems and aminoglycosides, while polymyxins show lower, though still clinically relevant, resistance levels (Figure 5A).

For *K. pneumoniae*, MDR biofilm-producing isolates showed high resistance rates to β-lactams and cephalosporins, in some cases reaching 100%. However, these findings should be interpreted with caution, as they may be influenced by the limited number of isolates analyzed. Resistance rates were moderately lower for carbapenems, aminoglycosides and fluoroquinolones. Interestingly, for the latter class, non-MDR non-biofilm-producing isolates displayed higher resistance percentages than MDR biofilm-producing strains, although this observation may reflect variability within the dataset rather than a consistent biological trend (Figure 5B).

*A. baumannii* exhibited resistance rates approaching 100% across multiple antibiotic classes, with polymyxins retaining partial activity (Figure 5C).

Similarly, *P. aeruginosa* showed elevated resistance rates across several classes, with comparatively lower resistance to aminoglycosides and polymyxins (Figure 5D).

### 3.7. Association Between MDR Status and Biofilm Production Capacity

**Figure 6:**
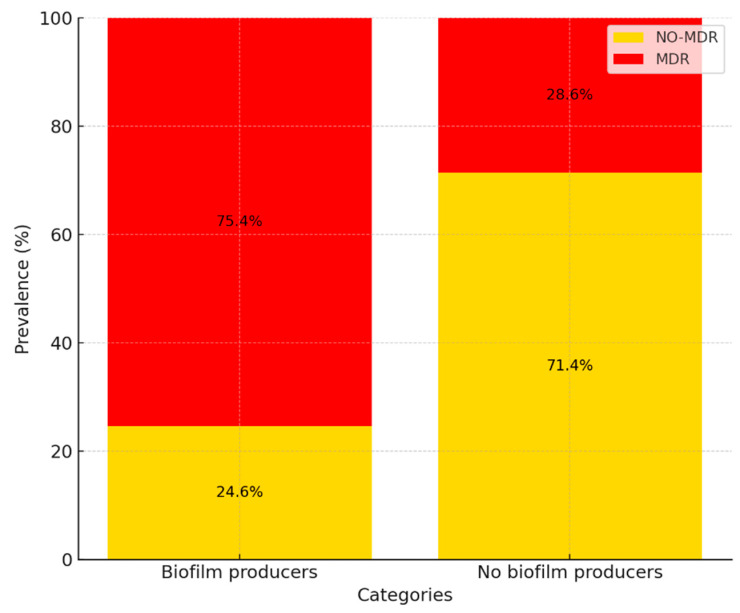
Prevalence of MDR and non-MDR strains among the different biofilm productions.

A statistically significant association emerged between MDR phenotype and biofilm production (*p* < 0.005; Figure 6).

### 3.8. Distribution by MDR Category and Bacterial Species

**Figure 7:**
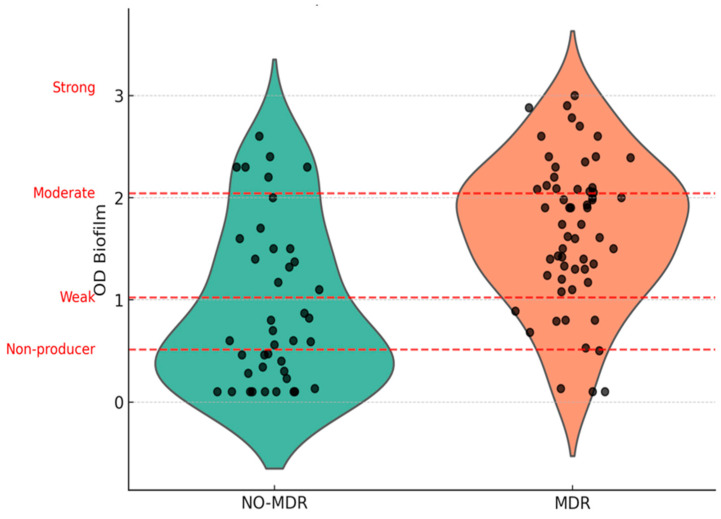
Biofilm optical density by MDR and non-MDR isolates. Most non-MDR isolates fall into the no-producer category (n = 21), while MDR isolates are within the moderate (n = 39) and strong (n = 26) biofilm producer categories. Non-producer: OD ≤ 0.51; Weak: 0.52 < OD < 1.02: Moderate: 1.03 < OD < 2.04; Strong: OD > 2.04. (The black dots indicate all the isolated units of the various devices).

**Figure 8:**
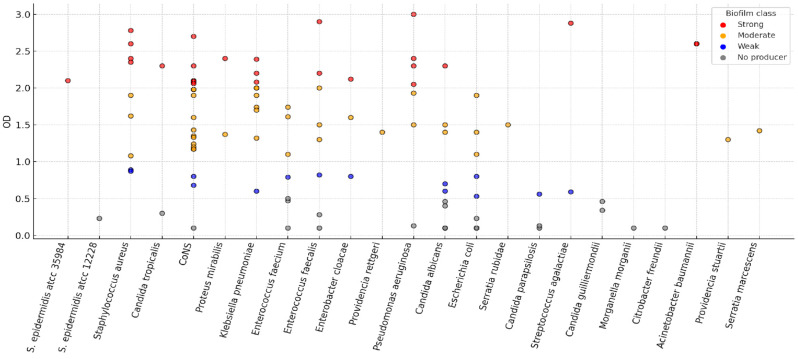
Distribution of biofilm optical density by microbial strains. Biofilm production varies across the different strains isolated and for some varies also in between the same species. Indeed, for CoNS, *E. faecium*, *P. aeruginosa*, and *C. albicans* isolates, biofilm production ranged from non-producers to strong producers, while for others, such as *A. baumannii*, all isolates were strong biofilm producers.

Detailed analysis by bacterial species as shown in Figure 7 and Figure 8, reveal consistent patterns favoring enhanced biofilm production in MDR strains. The majority of MDR isolates were distributed across “moderate to strong producer” classifications, whereas non-MDR counterparts predominantly occupied weak-producer and non-producer categories. These differences were supported by optical density measurements, showing a statistically significant difference between groups (*p* < 0.005).

### 3.9. Comparison of Biofilm Production for MDR Versus Non-MDR Strains by Species

**Figure 9:**
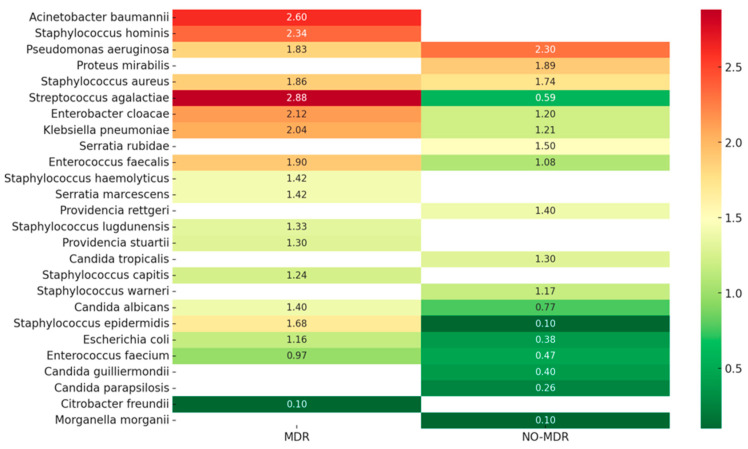
Different mean OD for bacterial strain in the MDR and non-MDR categories.

As shown in Figure 9, optical density (OD) values of biofilm production are reported for each bacterial species, with separate columns comparing MDR and non-MDR isolates. In almost all species examined (for example *Streptococcus agalactiae*, *Enterobacter cloacae*, *Klebsiella pneumoniae*), MDR isolates display higher OD values than their non-MDR counterparts, as reflected by the predominance of warmer colors in the MDR column and cooler tones in the non-MDR column. Species such as *Streptococcus agalactiae*, *Acinetobacter baumannii* and *Staphylococcus hominis* show the highest OD values among MDR strains, indicating a pronounced biofilm-forming capacity, whereas several non-MDR isolates exhibit reduced or negligible biofilm production.

### 3.10. Biofilm Production by Medical Device Type

**Figure 10:**
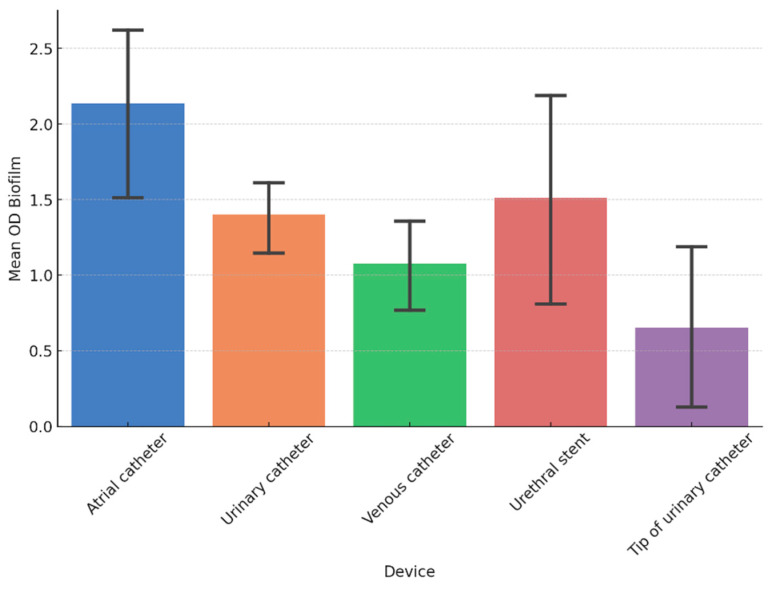
Average OD difference per device (only devices with 5 or more isolates). An ANOVA test was conducted to assess whether the variation in biofilm production between different medical devices was statistically significant (*p*-value = 0.028).

Only device categories with at least five isolates were included in this analysis, to ensure adequate microbiological representation and allow meaningful statistical comparisons of biofilm production among device types. As shown in Figure 10, biofilm production varied significantly by device type (*p* = 0.028), with atrial catheters and urethral stents showing higher mean OD values. Urethral stents showed similar biofilm production (1.51 ± 1.05). Urinary catheters exhibited an intermediate capacity to support biofilm formation (1.40 ± 0.79), while venous catheters generated a moderate amount of biofilm (1.08 ± 0.63). Finally, urinary catheter tips exhibited minimal biofilm production (0.65 ± 0.70), despite being associated with the same device type.

## 4. Discussion

The widespread use of medical devices (MDs) in modern healthcare has substantially improved patient outcomes but has simultaneously increased the incidence of device-related infections (DRIs) [14]. These infections represent a major clinical burden, as they are frequently associated with prolonged hospitalization, increased healthcare costs, and high rates of therapeutic failure. A key factor underlying the problematic nature of DRIs is the ability of microorganisms to form biofilms on device surfaces, including catheters, stents, and prosthetic implants. Biofilm-associated growth profoundly alters microbial physiology and represents a central determinant of antimicrobial tolerance through multiple mechanisms [15]. Compared with planktonic counterparts, biofilm-associated cells can exhibit dramatically increased resistance to antibiotics, in some cases up to 1000-fold higher [16]. This enhanced tolerance is multifactorial: the extracellular polymeric substance (EPS) matrix acts as a diffusion barrier limiting antibiotic penetration; the high cellular density promotes horizontal gene transfer and the dissemination of resistance determinants, such as *mecA* in methicillin-resistant staphylococci and carbapenemase-encoding genes in Enterobacterales; and the presence of metabolically inactive or slow-growing subpopulations further reduces antibiotic susceptibility [17].

In the present study, 100 clinical isolates were recovered from a heterogeneous collection of indwelling and implantable medical devices over a six-month period (January–June 2025). Within this cohort, coagulase-negative staphylococci (CoNS) represented the most frequently isolated group, followed by *K. pneumoniae*, *C. albicans*, and *S. aureus*. This distribution aligns with previous reports describing these organisms as common colonizers of medical devices [18,19]. However, it should be emphasized that the observed microbial distribution reflects the specific composition of the analyzed device cohort and should not be interpreted as representative of all device-associated infections.

The predominance of CoNS may be explained by their origin as skin commensals and their strong capacity for surface adhesion and biofilm formation, supported by genetic determinants such as the *icaADBC* operon, which encodes the synthesis of polysaccharide intercellular adhesin (PIA), a key mediator of intracellular aggregation and biofilm maturation [20]. Beyond their microbiological role as efficient colonizers of device surfaces, CoNS also raise important diagnostic and therapeutic questions in routine clinical practice, which are further discussed below. Similarly, the detection of *S. aureus*, *K. pneumoniae*, and *C. albicans* aligns with their recognized role in device-associated infections and persistence in biofilm communities [21]. The presence of polymicrobial colonization further highlights the ecological complexity of these infections.

Microorganisms within polymicrobial communities may establish synergistic or antagonistic interactions that influence biofilm maturation, extracellular matrix production, nutrient utilization, antimicrobial tolerance, and the exchange of resistance determinants [22]. According to Mishra et al., the principal biofilm-forming pathogens associated with medical devices include CoNS, *S. aureus*, *Enterococcus* spp., major Gram-negative opportunists such as *P. aeruginosa* and *K. pneumoniae*, and *Candida* species. The study also reports the occurrence of mono-, bi-, and even trimicrobial contamination patterns on device surfaces, further underscoring the polymicrobial nature and ecological complexity of many biofilm-associated infections [23]. Interactions between *Staphylococcus* spp., Enterobacterales, and *Candida* spp. have been shown to increase biofilm biomass and antimicrobial tolerance through mechanisms such as cross-protection, metabolic cooperation, and quorum-sensing modulation [24,25]. In particular, *Candida* and *Staphylococcus* interactions may result in enhanced invasiveness and resistance compared with monomicrobial infections [26]. These findings support the concept that device-associated infections may be better interpreted as structured polymicrobial ecosystems rather than strictly species events. According to Liao et al., interactions between *Enterobacteriaceae* family, *Enterococcus* spp., *Staphylococcus* spp., and fungal pathogens such as *Candida* spp. have been shown to enhance biofilm biomass and antimicrobial tolerance through mechanisms such as metabolic cooperation, cross-protection, and quorum-sensing modulation. These organisms are well recognized for their ability to adhere to indwelling medical devices and to form structured biofilms, which significantly enhance their persistence and tolerance to antimicrobial therapy [27]. Although the present study was not designed to investigate these mechanisms directly, such interactions may contribute to the persistence and complexity of device-associated microbial communities and should be explored in future studies [28].

A key finding of this study is the association between MDR phenotype and biofilm production, with MDR isolates showing a higher likelihood of biofilm formation compared with non-MDR isolates. This finding suggests that antimicrobial resistance and biofilm formation may represent interconnected adaptive strategies that contribute to microbial survival under antibiotic selective pressure. However, given the heterogeneity of microbial species and device types included in the analysis, this association should be interpreted with caution, as these variables may also influence biofilm formation. Further studies using multivariate analyses are needed to determine whether MDR independently predicts biofilm-forming capacity.

From a clinical perspective, these data highlight the limitations of conventional antimicrobial therapy in the absence of device removal. The persistence of MDR biofilm-producing strains underscores the need for integrated management strategies, including timely device replacement and the development of preventive approaches such as anti-adhesive surface modifications, antimicrobial-impregnated materials, quorum-sensing inhibitors, and biofilm-disrupting agents.

Overall, our results support the role of biofilm production as a key factor in the pathogenesis and clinical outcome of device-associated infections and suggest that it should be considered in both microbiological diagnostics and therapeutic decision-making.

The antimicrobial resistance profiles observed in this study are consistent with current epidemiological trends. High resistance among staphylococci suggests *S. aureus* further reflects its recognized role in prosthetic and catheter-related infections, where biofilm formation critically contributes to persistence and recurrence [29]. Among Gram-negative isolates, the high prevalence of *K. pneumoniae* is consistent with the increasing importance of Enterobacterales in healthcare-associated infections, especially in hospital settings characterized by high antibiotic selective pressure [30,31]. The detection of *C. albicans* confirms the role of fungal pathogens in device colonization, where biofilm development significantly enhances tolerance to antifungal agents and host immune responses [32].

Biofilm-mediated tolerance and conventional antimicrobial resistance represent distinct but synergistic mechanisms of bacterial survival. The resistance profiles identified in this study suggest that, beyond the protective matrix effect, chromosomally and plasmid-encoded resistance determinants further contribute to the persistence of these isolates in the clinical setting. High resistance rates to penicillin and oxacillin among staphylococci indicate a substantial prevalence of methicillin-resistant strains (MRSA and MR-CoNS), consistent with the widespread dissemination of the *mecA*-carrying staphylococcal cassette chromosome *mec* (*SCCmec*) in hospital environments [33,34]. The preserved activity of vancomycin and linezolid observed in this study is in agreement with European surveillance data, which still report relatively low resistance rates to these agents among staphylococci in most EU/EEA countries [35]. Nevertheless, the multidrug-resistant (MDR) profiles detected in species such as *S. haemolyticus* and *S. epidermidis* underscore their emerging role as reservoirs of resistance determinants [36,37,38]. Among enterococci, the more resistant phenotype observed in *E. faecium* compared with *E. faecalis* confirms global epidemiological trends, where *E. faecium* is frequently associated with multidrug resistance, including high-level resistance to ampicillin and aminoglycosides [39,40].

Comparative analysis with national surveillance data (AR-ISS) revealed significantly higher resistance rates among MDR biofilm-forming isolates, particularly among CoNS and *S. aureus*, where resistance to β-lactams and fluoroquinolones markedly exceeded reference values. These differences may reflect the specific characteristics of the study cohort and the limited number of isolates for some species [41]. Notably, MDR *S. aureus* biofilm-forming isolates showed resistance rates significantly higher than AR-ISS data, particularly for β-lactams and fluoroquinolones, reaching values up to 90–100% compared to the approximately 25% MRSA and 34–35% fluoroquinolone resistance reported in national reports. Increased resistance to macrolides was also evident, while aminoglycosides and glycopeptides maintained relatively lower levels of resistance, confirming their role as therapeutic options (Table 1).

A similar trend was observed in Gram-negative bacteria, with *Acinetobacter baumannii* and *P. aeruginosa* exhibiting high resistance rates to carbapenems and fluoroquinolones, consistent with their classification as critical priority pathogens [42,43,44]. For MDR and biofilm-forming strains of *A. baumannii*, only polymyxins retained partial activity. Similarly, *Pseudomonas aeruginosa* demonstrated a significant increase in resistance compared to surveillance data, particularly to aminoglycosides and fosfomycin, while polymyxins remained among the most active agents (Table 2) [41].

The coexistence of acquired resistance and biofilm-mediated tolerance in our isolates highlights the complex therapeutic challenge posed by these pathogens, particularly in device-related and persistent infections [45].

Regarding *K. pneumoniae*, elevated resistance to third- and fourth-generation cephalosporins and β-lactam/β-lactamase inhibitor combinations reflect the global dissemination of ESBL- and carbapenemase-producing strains. Within biofilms, these resistance mechanisms are further reinforced by limited antibiotic penetration, metabolic heterogeneity, and the presence of persister cells [46]. Notably, our results demonstrated a statistically significant association between MDR phenotype and biofilm production, with MDR isolates showing higher biofilm biomass compared to non-MDR counterparts. This contrasts with previous reports in which no significant association was observed. Nirwati et al. reported that 85.6% of *K. pneumoniae* clinical isolates were biofilm producers and 54.5% were multidrug-resistant (MDR); however, no statistically significant association was observed between MDR phenotype and biofilm-forming capacity [47]. In our results, MDR strains showed a markedly higher probability of being biofilm producers and greater mean optical density (OD) values compared to non-MDR isolates. Specifically, for *K. pneumoniae*, MDR isolates exhibited substantially greater biofilm production (mean OD ~2.04) compared to non-MDR strains (mean OD ~1.21). This discrepancy may reflect differences in study design, population, or analytical approaches.

Importantly, our findings support the hypothesis that biofilm formation may act as an amplifying factor for antimicrobial resistance in device-associated infections and complement previous studies that focused on single-species or single-device models by providing data from a heterogeneous device cohort [48,49].

Fungal isolates also demonstrated relevant biofilm-forming capacity. In *C. albicans*, MDR isolates exhibited higher biofilm production compared to non-MDR strains, consistent with previous studies. Silva et al. reported that biofilm-associated cells exhibit markedly reduced susceptibility to antifungal agents compared to their planktonic counterparts, primarily due to matrix-associated barriers, altered metabolic states, and the presence of persister cells [50]. Previous studies have demonstrated that *Candida* biofilm formation is associated with increased tolerance to fluconazole and other antifungal agents, contributing to persistent device-related infections (Table 3) [51]. These findings suggest that similar adaptive strategies may operate in fungal pathogens and emphasize the importance of antifungal susceptibility testing in device-related candidiasis. Furthermore, we have presented the antibiogram of those strains identified only once in a separate table (Table 4).

Finally, the significant variation in biofilm production across different device types highlights the role of material properties and local microenvironmental conditions in biofilm development. Factors such as surface roughness, chemical composition, protein adsorption, and fluid dynamics are known to influence microbial adhesion and subsequent biofilm maturation [52]. Differences observed between device categories suggest that these variables may contribute to variability in biofilm biomass and persistence, although they were not directly assessed in this study [52,53,54,55,56].

Our data also emphasizes the clinical relevance of CoNS on medical devices. The distinction between “contaminants” and true pathogens when CoNS are isolated from device-associated specimens requires careful discussion, since misclassification may lead either to unnecessary antibiotic use, favoring the emergence of drug-resistant bacteria, or to undertreatment with potentially severe consequences for patients [57]. The high incidence of CoNS observed on explanted devices in this study reinforces the need for accurate microbiological interpretation and adherence to clinical good practices, particularly when CoNS are associated with biofilm formation and multidrug-resistant profiles that often require second-line agents [58,59,60].

We also documented a high prevalence of methicillin-resistant strains (MRSA and MR-CoNS), paired with a preserved activity for vancomycin and linezolid. We then showed how our data aligns to the global epidemiological trends for enterococci and dissemination of ESBL- and carbapenemase-producing strains but differ for the resistance rates of *S. aureus* especially to β-lactams and fluoroquinolones. These results give an insight into our local epidemiology, which is informative for our own patients’ management, but is also important in the context of drug-resistant determinants in neighboring regions [61] and complement published data on resistance trends in *S. aureus* and CoNS from other clinical settings, such as ocular infections [62].

Lastly, to the best of our knowledge, only a limited number of studies have analyzed biofilm formation in relation to drug-resistant phenotypes across multiple microorganisms isolated from a broad spectrum of medical devices explanted from patients within a single facility. Previous reports with a similar design generally included fewer device categories and less microbial diversity, whereas our study combines a heterogeneous device panel (catheters, stents, catheter tips, prosthetic and fixation material) with a wide range of bacterial and fungal species.

By showing a significant variation in biofilm production across different device types, our results support further investigation of material properties and local microenvironments as determinants of biofilm development and persistence.

## 5. Conclusions

This study demonstrates a significant association between multidrug resistance (MDR) and enhanced biofilm-forming capacity in microorganisms isolated from medical devices, suggesting a potential synergistic contribution of these traits to microbial persistence in device-associated infections.

However, due to the observational design and heterogeneity of the cohort, this association must be interpreted with caution, as causality and full independence between MDR status and biofilm production cannot be established. Larger studies incorporating multivariate analyses will be required to clarify the relative contribution of microbial species, resistance profiles, and device-related factors and to assess the generalizability of our findings. In addition, the development of standardized biofilm susceptibility testing and clinically relevant experimental models will be essential to translate these insights into practice.

From a clinical perspective, our data underscores that device-associated infections often involve biofilm-producing MDR microorganisms, reinforcing the limitations of antimicrobial therapy, as biofilm architecture impairs drug penetration, sustains metabolically heterogeneous populations, and fosters persistent cell survival. These observations highlight the need for integrated management strategies that combine accurate microbiological diagnosis, careful interpretation of CoNS and other potential “contaminants”, and timely clinical decision-making. These strategies include timely device replacement and the development of preventive approaches such as anti-adhesive coatings, antimicrobial-impregnated materials, quorum-sensing inhibitors, and biofilm-disrupting agents.

Overall, addressing device-associated infections requires a multidisciplinary approach that integrates microbiology, materials science, infection control, and clinical management, and should be informed by local and regional epidemiological data on MDR and biofilm-producing pathogens. This integrated framework is essential to effectively address the intertwined challenges of biofilm formation and antimicrobial resistance in modern healthcare settings.

## Figures and Tables

**Table 1:** Antibiotic resistance profile of Gram-positive bacteria. (The values in the table represent the percentage).

Gram+	FA	AMC	AMP	PEN	CLI	DAP	ERY	GEN	IPM	KAN	LVX	LZD	OXA	RIF	STR	TEC	TET	TGC	SXT	VAN
*Enterococcus faecalis*	-	37.5	-	-	-	37.5	-	-	37.5	62.5	-	-	-	-	62.5	-	-	-	-	-
*Enterococcus faecium*	-	100	100	-	-	42.8	-	-	100	85.7	-	14.2	-	-	85.7	42.8	-	-	-	42.8
*Staphylococcus aureus*	-	-	-	88.8	77.7	11.1	44.4	11.1	-	-	77.7	11.1	66.6	11.1	-	33.3	22.2	-	11.1	33.3
*Staphylococcus capitis*	-	-	-	100	100	-	100	100	-	-	100	100	100	100	-	-	-	-	-	-
*Staphylococcus epidermidis*	33.3	-	-	90.9	58.3	8.3	83.3	50	-	-	100	-	83.3	58.3	-	25	75	8.3	41.6	8.3
*Staphylococcus haemolyticus*	66.6	-	-	66.6	66.6	33.3	100	66.6	-	-	100	-	66.6	66.6	-	100	66.6	-	66.6	33.3
*Staphylococcus hominis*	50	-	-	50	50	-	100	50	-	-	100	-	50	-	-	-	100	-	50	-
*Staphylococcus lugdunensis*	100	-	-	100	100	-	-	-	-	-	100	-	100	100	-	100	-	-	-	100
*Staphylococcus warneri*	-	-	-	-	-	-	100	-	-	-	100	-	-	-	-	-	-	-	-	-
*Streptococcus agalactiae*	-	-	-	-	100	-	100	-	-	-	50	-	-	100	-	-	100	-	-	-

Abbreviations: FA: Fusidic acid; AMC: Amoxicillin/Clavulanic acid; AMP: Ampicillin; PEN: Benzylpenicillin; CLI: Clindamycin; DAP: Daptomycin; ERY: Erythromycin; GEN: Gentamicin; IPM: Imipenem; KAN: Kanamycin; LVX: Levofloxacin; LZD: Linezolid; OXA: Oxacillin; RIF: Rifampicin; STR: Streptomycin; TEC: Teicoplanin; TET: Tetracycline; TGC: Tigecycline; SXT: Trimethoprim/Sulfamethoxazole; VAN: Vancomycin.

**Table 2:** Antibiotic resistance profile of Gram-negative bacteria. (The values in the table represent the percentage).

Gram-	AMK	AMC	FEP	CAZ	CZA	CTZ	CIP	COL	FOS	GEN	LVX	MEM	MVB	TZP	SXT
*Acinetobacter baumannii*	100	100	-	-	-	-	100	50	-	100	100	100	-	50	100
*Citrobacter freundii*	-	100	100	100	100	100	100	-	100	-	-	-	-	100	-
*Enterobacter cloacae*	-	100	33.3	33.3	33.3	33.3	-	33.3	-	-	-	33.3	33.3	33.3	-
*Escherichia coli*	-	50	62.5	62.5	25	25	50	12.5	-	25	-	25	25	25	-
*Klebsiella pneumoniae*	-	80	80	80	30	70	50	-	50	40	-	50	30	60	-
*Morganella morganii*	-	100	-	-	-	-	-	100	-	-	-	-	-	-	-
*Proteus mirabilis*	-	100	-	-	-	-	-	100	-	-	-	-	-	50	-
*Providencia rettgeri*	-	100	-	-	-	-	-	100	-	-	-	-	-	-	-
*Providencia stuartii*	-	100	-	100	-	-	100	100	100	100	-	-	-	-	-
*Pseudomonas aeruginosa*	-	100	83.3	83.3	33.3	16.6	83.3	40	83.3	83.3	-	-	-	83.3	-
*Serratia marcescens*	-	-	-	100	-	-	100	100	100	100	-	-	-	-	-
*Serratia rubidae*	100	-	-	-	-	-	100	100	-	-	-	-	-	-	-

Abbreviations: AMK: Amikacin; AMC: Amoxicillin/Clavulanic acid; FEP: Cefepime; CAZ: Ceftazidime; CZA: Ceftazidime/Avibactam; CTZ: Ceftolozane/Tazobactam; CIP: Ciprofloxacin; COL: Colistin; FOS: Fosfomycin; GEN: Gentamicin; LVX: Levofloxacin; MEM: Meropenem; MVB: Meropenem/Vaborbactam; TZP: Piperacillin/Tazobact; SXT: Trimethoprim/Sulfamethoxazole.

**Table 3:** Antibiotic resistance profile of *Candida* species. (The values in the table represent the percentage).

Yeast	CAS	FLU	MICA	VORI
*Candida albicans*	-	22.2	11.1	22.2
*Candida guilliermondii*	-	-	-	-
*Candida parapsilosis*	-	33.3	-	33.3
*Candida Tropicalis*	-	-	-	-

Abbreviations: CAS: Caspofungin; FLU: Fluconazole; MICA: Micafungin; VORI: Voriconazole.

**Table 4:** Antimicrobial susceptibility profiles of infrequently isolated Gram-negative species (*n* = 1 isolate per species).

Gram-	AMK	AMC	FEP	CAZ	CZA	CTZ	CIP	COL	FOS	GEN	LVX	MEM	MVB	TZP	SXT
*Citrobacter freundii*	-	100	100	100	100	100	100	-	100	-	-	-	-	100	-
*Morganella morganii*	-	100	-	-	-	-	-	100	-	-	-	-	-	-	-
*Providencia rettgeri*	-	100	-	-	-	-	-	100	-	-	-	-	-	-	-
*Providencia stuartii*	-	100	-	100	-	-	100	100	100	100	-	-	-	-	-
*Serratia marcescens*	-	-	-	100	-	-	100	100	100	100	-	-	-	-	-
*Serratia rubidae*	100	-	-	-	-	-	100	100	-	-	-	-	-	-	-

Abbreviations: AMC: Amoxicillin/Clavulanic acid; AMK: Amikacin; CAZ: Ceftazidime; CTZ: Ceftolozane/Tazobactam; CIP: Ciprofloxacin; CZA: Ceftazidime/Avibactam; COL: Colistin; FEP: Cefepime; FOS: Fosfomycin; GEN: Gentamicin; LVX: Levofloxacin; MEM: Meropenem; MVB: Meropenem/Vaborbactam; TZP: Piperacillin/Tazobact; SXT: Trimethoprim/Sulfamethoxazole.

## Data Availability

The original contributions presented in the study are included in the article, further inquiries can be directed to the corresponding author.

## References

[1] Chukamnerd A., Saipetch N., Singkhamanan K., Ingviya N., Assanangkornchai N., Surachat K., Chusri S. (2024). Association of Biofilm Formation, Antimicrobial Resistance, Clinical Characteristics, and Clinical Outcomes among Acinetobacter Baumannii Isolates from Patients with Ventilator-Associated Pneumonia. Clin. Respir. J..

[2] Bouhrour N., Nibbering P.H., Bendali F. (2024). Medical Device-Associated Biofilm Infections and Multidrug-Resistant Pathogens. Pathogens.

[3] Donlan R.M. (2001). Biofilm Formation: A Clinically Relevant Microbiological Process. Clin. Infect. Dis..

[4] de Kievit T.R. (2009). Quorum Sensing in *Pseudomonas Aeruginosa* Biofilms. Environ. Microbiol..

[5] Asokan S., Pandey R.K., Jalil M.A., Alhussen S.K.A., Yousif S.I.A., Abbas R.K., Vijayan S., Rajeswary D., Jacob T., Atiyah M.M. (2026). Biofilm Associated Infections on Medical Devices: Pathogenesis, Diagnostic Challenges, and Control Strategies. Microbe.

[6] Dadi N.C.T., Radochová B., Vargová J., Bujdáková H. (2021). Impact of Healthcare-Associated Infections Connected to Medical Devices—An Update. Microorganisms.

[7] Bryers J.D. (2008). Medical Biofilms. Biotechnol. Bioeng..

[8] Gahlot R., Nigam C., Kumar V., Yadav G., Anupurba S., Gahlot R., Nigam C., Kumar V., Yadav G., Anupurba S. (2014). Catheter-Related Bloodstream Infections. Int. J. Crit. Illn. Inj. Sci..

[9] Donlan R.M. (2001). Biofilms and Device-Associated Infections. Emerg. Infect. Dis..

[10] Stepanovic S., Vukovic D., Dakic I., Savic B., Svabic-Vlahovic M. (2000). A Modified Microtiter-Plate Test for Quantification of Staphylococcal Biofilm Formation. J. Microbiol. Methods.

[11] Catania A.M., Di Ciccio P., Ferrocino I., Civera T., Cannizzo F.T., Dalmasso A. (2023). Evaluation of the Biofilm-Forming Ability and Molecular Characterization of Dairy Bacillus Spp. Isolates. Front. Cell. Infect. Microbiol..

[12] Lutfi L.L., Shaaban M.I., Elshaer S.L. (2024). Vitamin D and Vitamin K1 as Novel Inhibitors of Biofilm in Gram-Negative Bacteria. BMC Microbiol..

[13] R Core Team (2025). R: A Language and Environment for Statistical Computing.

[14] Garvey M. (2023). Medical Device-Associated Healthcare Infections: Sterilization and the Potential of Novel Biological Approaches to Ensure Patient Safety. Int. J. Mol. Sci..

[15] Garcia L.S., Roque-Borda C.A., Pavan F.R., Chorilli M. (2026). Biofilm Associated Persistence and Drug Tolerance in Mycobacteria Within Host Microenvironments. APMIS.

[16] Hindieh P., Yaghi J., Assaf J.C., Chokr A., Atoui A., Tzenios N., Louka N., Khoury A.E. (2025). Emerging Multimodal Strategies for Bacterial Biofilm Eradication: A Comprehensive Review. Microorganisms.

[17] Uruén C., Chopo-Escuin G., Tommassen J., Mainar-Jaime R.C., Arenas J. (2020). Biofilms as Promoters of Bacterial Antibiotic Resistance and Tolerance. Antibiotics.

[18] Otto M. (2018). Staphylococcal Biofilms. Microbiol. Spectr..

[19] Becker K., Heilmann C., Peters G. (2014). Coagulase-Negative Staphylococci. Clin. Microbiol. Rev..

[20] Arciola C.R., Campoccia D., Montanaro L. (2018). Implant Infections: Adhesion, Biofilm Formation and Immune Evasion. Nat. Rev. Microbiol..

[21] Folliero V., Franci G., Dell’Annunziata F., Giugliano R., Foglia F., Sperlongano R., De Filippis A., Finamore E., Galdiero M. (2021). Evaluation of Antibiotic Resistance and Biofilm Production among Clinical Strain Isolated from Medical Devices. Int. J. Microbiol..

[22] Hall-Stoodley L., Costerton J.W., Stoodley P. (2004). Bacterial Biofilms: From the Natural Environment to Infectious Diseases. Nat. Rev. Microbiol..

[23] Mishra A., Aggarwal A., Khan F. (2024). Medical Device-Associated Infections Caused by Biofilm-Forming Microbial Pathogens and Controlling Strategies. Antibiotics.

[24] Peters B.M., Jabra-Rizk M.A., O’May G.A., Costerton J.W., Shirtliff M.E. (2012). Polymicrobial Interactions: Impact on Pathogenesis and Human Disease. Clin. Microbiol. Rev..

[25] Allison D.L., Willems H.M.E., Jayatilake J.a.M.S., Bruno V.M., Peters B.M., Shirtliff M.E. (2016). Candida-Bacteria Interactions: Their Impact on Human Disease. Microbiol. Spectr..

[26] Harriott M.M., Noverr M.C. (2011). Importance of Candida-Bacterial Polymicrobial Biofilms in Disease. Trends Microbiol..

[27] Liao W.-C., Chung W.-S., Lo Y.-C., Shih W.-H., Chou C.-H., Chen C.-Y., Tu C.-Y., Ho M.-W. (2022). Changing Epidemiology and Prognosis of Nosocomial Bloodstream Infection: A Single-Center Retrospective Study in Taiwan. J. Microbiol. Immunol. Infect..

[28] Lee K.W.K., Periasamy S., Mukherjee M., Xie C., Kjelleberg S., Rice S.A. (2014). Biofilm Development and Enhanced Stress Resistance of a Model, Mixed-Species Community Biofilm. ISME J..

[29] Tong S.Y.C., Davis J.S., Eichenberger E., Holland T.L., Fowler V.G. (2015). Staphylococcus Aureus Infections: Epidemiology, Pathophysiology, Clinical Manifestations, and Management. Clin. Microbiol. Rev..

[30] Pitout J.D.D., Laupland K.B. (2008). Extended-spectrum β-lactamase-producing Enterobacteriaceae: An emerging public-health concern. Lancet Infect. Dis..

[31] Alcántar-Curiel M.D., Ledezma-Escalante C.A., Jarillo-Quijada M.D., Gayosso-Vázquez C., Morfín-Otero R., Rodríguez-Noriega E., Cedillo-Ramírez M.L., Santos-Preciado J.I., Girón J.A. (2018). Association of Antibiotic Resistance, Cell Adherence, and Biofilm Production with the Endemicity of Nosocomial Klebsiella Pneumoniae. BioMed Res. Int..

[32] Taff H.T., Mitchell K.F., Edward J.A., Andes D.R. (2013). Mechanisms of Candida Biofilm Drug Resistance. Future Microbiol..

[33] Lundin P.M., Fiser B.L., Blackledge M.S., Pickett H.L., Copeland A.L. (2022). Functionalized Self-Assembled Monolayers: Versatile Strategies to Combat Bacterial Biofilm Formation. Pharmaceutics.

[34] Abdelwahab M.A., Amer W.H., Elsharawy D., Elkolaly R.M., Helal R.A.E.F., El Malla D.A., Elfeky Y.G., Bedair H.A., Amer R.S., Abd-Elmonsef M.E. (2023). Phenotypic and Genotypic Characterization of Methicillin Resistance in Staphylococci Isolated from an Egyptian University Hospital. Pathogens.

[35] Vitiello A., Zovi A., Sabbatucci M., Fabbro E., Ricciardi W., Villani L., Rezza G., Boccellino M., Capuano A., Rossi F. (2026). Intervention studies and antimicrobial resistance in Italy: An overview of the latest evidence. J. Glob. Antimicrob. Resist..

[36] Siciliano V., Passerotto R.A., Chiuchiarelli M., Leanza G.M., Ojetti V. (2023). Difficult-to-Treat Pathogens: A Review on the Management of Multidrug-Resistant Staphylococcus Epidermidis. Life.

[37] Silva P.V., Cruz R.S., Keim L.S., de Paula G.R., Carvalho B.T.F., Coelho L.R., Carvalho M.C.d.S., da Rosa J.M.C., Figueiredo A.M.S., Teixeira L.A. (2013). The Antimicrobial Susceptibility, Biofilm Formation and Genotypic Profiles of Staphylococcus Haemolyticus from Bloodstream Infections. Mem. Inst. Oswaldo Cruz.

[38] Huang C., Moradi S., Sholeh M., Tabaei F.M., Lai T., Tan B., Meng J., Azizian K. (2025). Global Trends in Antimicrobial Resistance of Enterococcus Faecium: A Systematic Review and Meta-Analysis of Clinical Isolates. Front. Pharmacol..

[39] Jabbari F., Nikoohemmat M., Ahmadian M., Akhgarzad A., Ebrahimi N., Javandoust Gharehbagh F., Alavi Darazam I. (2025). Antimicrobial Resistance Pattern of Enterococcus Species among Clinical Isolates in Iran: A Systematic Review and Meta-Analysis. J. Infect. Public Health.

[40] AR-ISS: Sorveglianza Nazionale dell’Antibiotico-Resistenza. https://www.iss.it/documents/20126/10087877/RIS-5_2025.pdf/7214a63d-9e5a-f022-2d9e-074200c2fbdf?t=1764685261748.

[41] Boutzoukas A., Doi Y. (2025). The global epidemiology of carbapenem-resistant *Acinetobacter baumannii*. JAC Antimicrob. Resist..

[42] Antochevis L.C., Wilhelm C.M., Arns B., Sganzerla D., Sudbrack L.O., Nogueira T.C.R.L., Guzman R.D., Martins A.S., Cappa D.S., Dos Santos Â.C. (2025). ASCENSION Study Group. World Health Organization Priority Antimicrobial Resistance in Enterobacterales, Acinetobacter Baumannii, Pseudomonas Aeruginosa, Staphylococcus Aureus and Enterococcus Faecium Healthcare-Associated Bloodstream Infections in Brazil (ASCENSION): A Prospective, Multicentre, Observational Study. Lancet Reg. Health Am..

[43] Yin R., Cheng J., Wang J., Li P., Lin J. (2022). Treatment of Pseudomonas Aeruginosa Infectious Biofilms: Challenges and Strategies. Front. Microbiol..

[44] Ciofu O., Tolker-Nielsen T. (2019). Tolerance and Resistance of Pseudomonas Aeruginosa Biofilms to Antimicrobial Agents-How P. Aeruginosa Can Escape Antibiotics. Front. Microbiol..

[45] Peirano G., Pitout J.D.D. (2019). Extended-Spectrum β-Lactamase-Producing Enterobacteriaceae: Update on Molecular Epidemiology and Treatment Options. Drugs.

[46] Qureshi K.A., Fahmy N.A., Parvez A., Almahasheer H., Permatasari D., Jaremko M., Abdallah E.M. (2026). Biofilms and Antimicrobial Resistance: Mechanisms, Clinical Implications, and Emerging Interventions. Chem. Biodivers..

[47] Nirwati H., Sinanjung K., Fahrunissa F., Wijaya F., Napitupulu S., Hati V.P., Hakim M.S., Meliala A., Aman A.T., Nuryastuti T. (2019). Biofilm Formation and Antibiotic Resistance of Klebsiella Pneumoniae Isolated from Clinical Samples in a Tertiary Care Hospital, Klaten, Indonesia. BMC Proc..

[48] Mishra S.K., Basukala P., Basukala O., Parajuli K., Pokhrel B.M., Rijal B.P. (2015). Detection of biofilm production and antibiotic resistance pattern in clinical isolates from indwelling medical devices. Curr. Microbiol..

[49] Gogoi M., Sharma A., Hazarika N.K. (2015). Biofilm formation by bacterial isolates from patients on indwelling medical devices. Indian J. Med. Microbiol..

[50] Silva S., Rodrigues C.F., Araújo D., Rodrigues M.E., Henriques M. (2017). Candida Species Biofilms’ Antifungal Resistance. J. Fungi.

[51] Squitieri D., Rizzo S., Torelli R., Mariotti M., Sanguinetti M., Cacaci M., Bugli F. (2025). Studying Candida Biofilms Across Species: Experimental Models, Structural Diversity, and Clinical Implications. Pharmaceuticals.

[52] Krsmanovic M., Biswas D., Ali H., Kumar A., Ghosh R., Dickerson A.K. (2021). Hydrodynamics and Surface Properties Influence Biofilm Proliferation. Adv. Colloid Interface Sci..

[53] Rajaramon S., Shanmugam K., Dandela R., Solomon A.P. (2023). Emerging Evidence-Based Innovative Approaches to Control Catheter-Associated Urinary Tract Infection: A Review. Front. Cell. Infect. Microbiol..

[54] Chadha J., Thakur N., Chhibber S., Harjai K. (2024). A Comprehensive Status Update on Modification of Foley Catheter to Combat Catheter-Associated Urinary Tract Infections and Microbial Biofilms. Crit. Rev. Microbiol..

[55] Ionescu A.C., Brambilla E., Sighinolfi M.C., Mattina R. (2021). A New Urinary Catheter Design Reduces In-Vitro Biofilm Formation by Influencing Hydrodynamics. J. Hosp. Infect..

[56] Souza J.G.S., Bertolini M., Liu J., Nagay B.E., Martins R., Costa R.C., Brunson J.C., Shibli J., Figueiredo L.C., Dongari-Bagtzoglou A. (2025). Exploring the Impact of Biotic and Abiotic Surfaces on Protein Binding Modulation and Bacteria Attachment: Integrating Biological and Mathematical Approaches. ACS Nano.

[57] Michels R., Last K., Becker S.L., Papan C. (2021). Update on Coagulase-Negative Staphylococci-What the Clinician Should Know. Microorganisms.

[58] Al-Ramlawi A.H., Al-adini S.O., Shaqoura A.S., Al-Mougher M.M., Khadoura K.J. (2026). Microbial contamination and infection control measures: Experience of a medical complex. J. Umm Al-Qura Univ. Med. Sci..

[59] Murphy S.C., Russell S.P., Harty J.A., O’Loughlin P. (2025). Microbiologic features of prosthetic joint infections at a tertiary referral orthopaedic unit. Ir. J. Med. Sci..

[60] Szabóová T., Gregová G., Király J., Daçovà N., Hajdučková V., Hudecová P., Hisirová S., Polan P., Lovayová V. (2026). Prevalence of Biofilm-Forming and Antibiotic-Resistant Coagulase-Negative Staphylococci Isolated from Hospitalized Patients in an Orthopedic Clinic. Pathogens.

[61] Alenzi S.L.O., Alharthi A.H., Alnefaie R.A., Alsaadi O., Albugami A.N.M., Alhusini K.M., Alanazi F.S., ALAnazi F.H.K., Alkhayyat A.A. (2026). The Global Epidemiology of Antimicrobial Resistance: Trends, Determinants, and Public Health Implications. Cureus.

[62] Petrillo F., Pignataro D., Maria Di Lella F., Reibaldi M., Fallico M., Castellino N., Parisi G., Trotta M.C., D’Amico M., Santella B. (2021). Antimicrobial Susceptibility Patterns and Resistance Trends of Staphylococcus aureus and Coagulase-Negative Staphylococci Strains Isolated from Ocular Infections. Antibiotics.

